# A phase space model of a Versa HD linear accelerator for application to Monte Carlo dose calculation in a real‐time adaptive workflow

**DOI:** 10.1002/acm2.13663

**Published:** 2022-06-14

**Authors:** James L. Bedford, Rahul Nilawar, Simeon Nill, Uwe Oelfke

**Affiliations:** ^1^ Joint Department of Physics The Institute of Cancer Research and The Royal Marsden NHS Foundation Trust London UK

**Keywords:** dose calculation, Monte Carlo simulation, phase space

## Abstract

**Purpose:**

This study aims to develop and validate a simple geometric model of the accelerator head, from which a particle phase space can be calculated for application to fast Monte Carlo dose calculation in real‐time adaptive photon radiotherapy. With this objective in view, the study investigates whether the phase space model can facilitate dose calculations which are compatible with those of a commercial treatment planning system, for convenient interoperability.

**Materials and methods:**

A dual‐source model of the head of a Versa HD accelerator (Elekta AB, Stockholm, Sweden) was created. The model used parameters chosen to be compatible with those of 6‐MV flattened and 6‐MV flattening filter‐free photon beams in the RayStation treatment planning system (RaySearch Laboratories, Stockholm, Sweden). The phase space model was used to calculate a photon phase space for several treatment plans, and the resulting phase space was applied to the Dose Planning Method (DPM) Monte Carlo dose calculation algorithm. Simple fields and intensity‐modulated radiation therapy (IMRT) treatment plans for prostate and lung were calculated for benchmarking purposes and compared with the convolution‐superposition dose calculation within RayStation.

**Results:**

For simple square fields in a water phantom, the calculated dose distribution agrees to within ±2% with that from the commercial treatment planning system, except in the buildup region, where the DPM code does not model the electron contamination. For IMRT plans of prostate and lung, agreements of ±2% and ±6%, respectively, are found, with slightly larger differences in the high dose gradients.

**Conclusions:**

The phase space model presented allows convenient calculation of a phase space for application to Monte Carlo dose calculation, with straightforward translation of beam parameters from the RayStation beam model. This provides a basis on which to develop dose calculation in a real‐time adaptive setting.

## 1 INTRODUCTION

The starting point of any dose calculation using Monte Carlo simulation is a phase space of particles exiting the head of the linear accelerator. The phase space is a list of the positions, directions, energies, and numbers of particles passing through a plane below the accelerator head.^1^, ^2^, ^3^ This phase space is dependent upon the geometry and settings of the accelerator head and the multileaf collimator. It can be generated by Monte Carlo simulation of radiation transport through the components in the head of the accelerator, such as by the BEAM^4^, ^5^ or MCNP code,^6^ but this is too slow to be clinically useful.

The alternative is to use an empirical model of the linear accelerator head, so that the phase space can be calculated relatively simply for each treatment field.^7^, ^8^ This is the approach that is used by deterministic dose calculations such as convolution‐superposition, although in this case the model is used to produce fluence rather than explicitly defining the individual particles.^9^ An empirical model of the accelerator head has also been used in the context of Monte Carlo simulation for some time. For example, Fippel et al.^10^ use two Gaussian‐shaped photon sources to generate fluence distributions for rectangular fields in conjunction with the XVMC Monte Carlo code. This model is then applied by Sikora et al.^11^ to a Beam Modulator treatment head (Elekta AB, Stockholm, Sweden).

Another common approach is to use Monte Carlo simulation to produce phase space files and then to extract information from these in a form which can be used for various collimator positions, usually with the aid of an empirical model.^12^, ^13^ Fix et al.^14^ use two sources to produce a simple phase space model in which the energy spectrum of the particles is varied according to field size. A further work uses 12 sources to model the main components of the linear accelerator head and apply the resulting phase space to the GEANT Monte Carlo code, showing good agreement with measured data for a Clinac 2300 accelerator (Varian Medical Systems, Palo Alto, CA).^15^ Individual sources in a three‐source model are also analyzed separately, so as to ensure appropriate contributions.^16^

More recently, Aboulbanine et al.^17^ model the current generation of linear accelerators using a phase space model consisting of primary and scatter components, with each of the scatter components being modeled in a customized manner. They apply the model to 6 and 10 MV beams from an Elekta Precise head and to the 6 MV beam from a Varian Truebeam accelerator. A more detailed model of a multileaf collimator allows for fast calculation of IMRT fields in the case of Elekta and Varian accelerators.^18^ These works show that accurate modeling of the linear accelerator head to produce a deterministic phase space is possible. Similar results are also obtained in the field of particle therapy.^19^, ^20^

There is currently considerable interest in fast dose calculation for application to dose reconstruction during adaptive radiotherapy.^21^ The goal of this field of research is to be able to display the dose distribution that is being delivered to the patient in near real time, as the patient is being treated, based on real‐time imaging systems and either a prior or adaptive treatment plan. Potentially, as the patient's state changes, the imaging system can measure the three‐dimensional form of the patient, the tumor can be visualized, the treatment plan adapted as necessary to track the tumor, and the delivered dose reconstructed.^22^ Such dose reconstruction requires that the treatment plan be calculated very fast, but also with significant accuracy. For this reason, an accurate but efficient phase space model is of increasing importance.

This paper therefore describes a simple but accurate phase space model for application to fast adaptive Monte Carlo dose calculation. For convenience of application, the parameters required for the model are designed to be compatible with those required for the photon beam model used by RayStation v10 (RaySearch Laboratories, Stockholm, Sweden).^23^ The resulting phase space is applied to the Monte Carlo code Dose Planning Method (DPM).^24^ A comparison is then made for various simple fields with the convolution‐superposition dose calculation used by RayStation. Finally, the same comparison is made for IMRT treatment plans of prostate and lung.

## 2 METHODS

### 2.1 Phase space model

**FIGURE 1:**
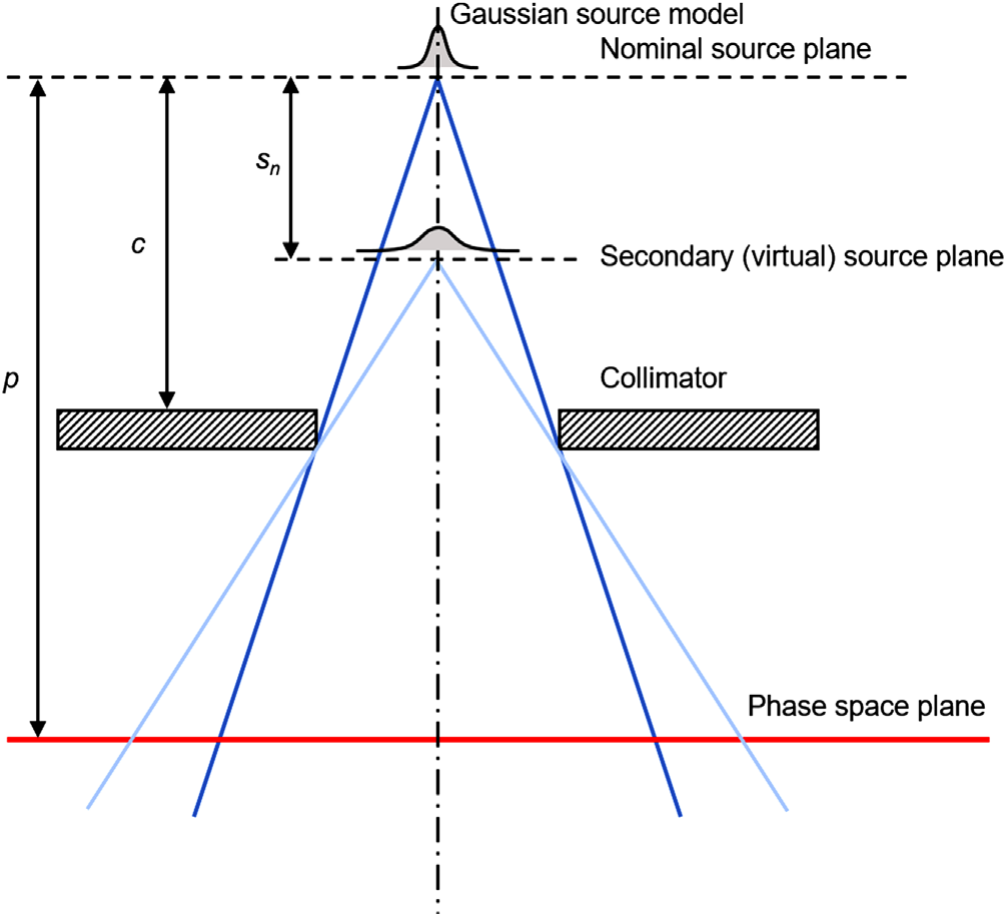
Schematic diagram of the phase space model for the case of two sources

For this study, the 6 MV beam of a Versa HD linear accelerator (Elekta AB, Stockholm, Sweden) was used.^25^ Both flattened and flattening filter‐free (FFF) beams were considered. The phase space model, illustrated schematically in Figure 1, was a generalized multiple‐source model consisting of a number of Gaussian‐shaped sources located on the central axis of the beam. In this work, two sources were used, one at the nominal source position of the accelerator and the second at the position of the flattening filter. Two sources were also used for the FFF beams so as to adequately model the scatter from the primary collimator. This section describes the theoretical basis of the phase space model, while Section 2.2 describes the generation of practical values.

Specifying particle positions and directions in the phase space required the use of a coordinate system. The origin of this coordinate system was at the nominal source of the beam (i.e., at the tungsten target), the *x*‐axis was directed orthogonally to the central axis in the same direction as defined by IEC61217, the *y*‐axis was directed towards the foot of the couch, and the *z*‐axis was directed along the central axis of the beam, towards the patient. In other words, the coordinate system was equivalent to the IEC61217 standard but rotated 180° about the *x*‐axis. This coordinate system was found to be the simplest when handling multiple sources in the beam axis. The distance from the nominal source of the beam to the isocenter was taken to be *d*. A rectangular grid was defined in the isocentric plane, whose grid points were indexed by *i* in the *x*‐direction and *j* in the *y*‐direction. The position of a grid point in the beam's eye view at the isocentric distance was given by:

(1a) $$x_{dij}=g_{x}+i\delta_{x}\left(i=0\text{…}I-1\right),$$

(1b) $$y_{dij}=g_{y}+j\delta_{y}\left(j=0\text{…}J-1\right),$$

(1c) $$z_{dij}=d,$$

where *g_x_* and *g_y_* were the starting coordinates of the edge of the grid in the *x*‐ and *y*‐directions, respectively; *δx* and *δy* were the grid resolutions in the *x*‐ and *y*‐directions, respectively.

The *N* sources of the beam model were located at positions *s_n_* (*n* = 1…*N*) from the origin, and the plane of the phase space was located a distance *p* from the origin, so that the grid points of Equation (1) projected to a position on the phase space plane of:

(2a) $$x_{pij}=\frac{p-s_{n}}{d-s_{n}}x_{dij},$$

(2b) $$y_{pij}=\frac{p-s_{n}}{d-s_{n}}y_{dij},$$

(2c) $$z_{pij}=p.$$

The distance from virtual source *n* to this point in the phase space was given by:

(3) $$r_{pij}=\sqrt{x_{pij}^{2}+y_{pij}^{2}+{\left(z_{pij}-s_{n}\right)}^{2}}.$$

At each of these locations in the phase space plane, a particle source was created, with position coordinates $\left(x_{pij},\;y_{pij},\;z_{pij}\right)$ and unit direction vector given by:

(4a) $${\hat{x}}_{pij}=\frac{x_{pij}}{r_{pij}},$$

(4b) $${\hat{y}}_{pij}=\frac{y_{pij}}{r_{pij}},$$

(4c) $$\;{\hat{z}}_{pij}=\frac{z_{pij}-s_{n}}{r_{pij}}.$$

**FIGURE 2:**
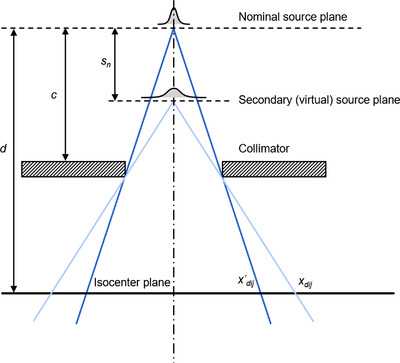
Transformation of coordinates from the secondary divergent system to the primary divergent system for comparison with collimator and fluence settings. To determine whether point (*i*, *j*) with position *x_dij_* lies within the beam aperture, the position $\frac{c-s_{n}}{d-s_{n}}x_{dij}$of the grid point at the collimator plane is calculated. However, the collimator setting is expressed at isocenter according to divergence given by the primary source, so the calculated coordinate must be scaled by *d*/*c*, giving $x_{dij}^{\prime }=\frac{d(c-s_{n})}{c(d-s_{n})}x_{dij}$. Diagram not to scale

Equations (2) and (4) defined the position and direction of the particles. The next step was to calculate the particle fluence. This required the use of quantities such as primary fluence and collimator position, which were tabulated in terms of off‐axis position at the isocenter plane. For example, the collimator position actually referred to the location of the collimator in the accelerator head, but its position was defined at the isocenter plane. The divergent projection used to relate the actual position of the component and its position at the isocenter plane was always constructed from the primary source, even for secondary sources (Figure 2), giving rise to a further set of coordinates at the isocenter plane:

(5a) $$x_{dij}^{\prime }=\frac{d\left(c-s_{n}\right)}{c\left(d-s_{n}\right)}x_{dij},$$

(5b) $$y_{dij}^{\prime }=\frac{d\left(c-s_{n}\right)}{c\left(d-s_{n}\right)}y_{dij},$$

(5c) $$z_{dij}^{\prime }=d.$$

The off‐axis position of these grid points is given by:

(6) $$r{{}^{\prime }}_{\mathrm{dij}}=\sqrt{x{{}^{\prime }}_{\mathrm{dij}}^{2}+y{{}^{\prime }}_{\mathrm{dij}}^{2}}.$$

The emitted fluence was calculated by taking the primary fluence, $\varphi ({r^{\prime }}_{dij})$, and modulating it by the beam aperture, $\omega ({x^{\prime }}_{dij},{y^{\prime }}_{dij},{z^{\prime }}_{dij})$. This latter variable had a value of unity if the point $\left({x^{\prime }}_{dij},{y^{\prime }}_{dij},{z^{\prime }}_{dij}\right)$ lay in the aperture defined by the jaws and the multileaf collimator, and zero otherwise. For the Versa HD accelerator head,^25^ the aperture was modeled using the variable *y*‐jaws (IEC61217) and the 160 leaves of the MLC, with their 5 mm spacing at isocenter. The *x*‐jaws of the Versa HD head were fixed at ±200 mm, so were not used in this work. Note that all of $\left({x^{\prime }}_{dij},{y^{\prime }}_{dij},{z^{\prime }}_{dij}\right)$, $\omega ({x^{\prime }}_{dij},{y^{\prime }}_{dij},{z^{\prime }}_{dij})$, and the jaw and MLC settings were defined at the isocenter plane. Facility was also provided for the representation of MLC transmission, but as the transmission of the Versa HD MLC was very low,^25^ the transmission value was set to zero for this work. The fluence was then given by:

(7) $$\Phi_{\mathrm{pij}}=\varphi \left({r^{\prime }}_{\mathrm{dij}}\right)\omega \left({x^{\prime }}_{\mathrm{dij}},{y^{\prime }}_{\mathrm{dij}},{z^{\prime }}_{\mathrm{dij}}\right).$$

**FIGURE 3:**
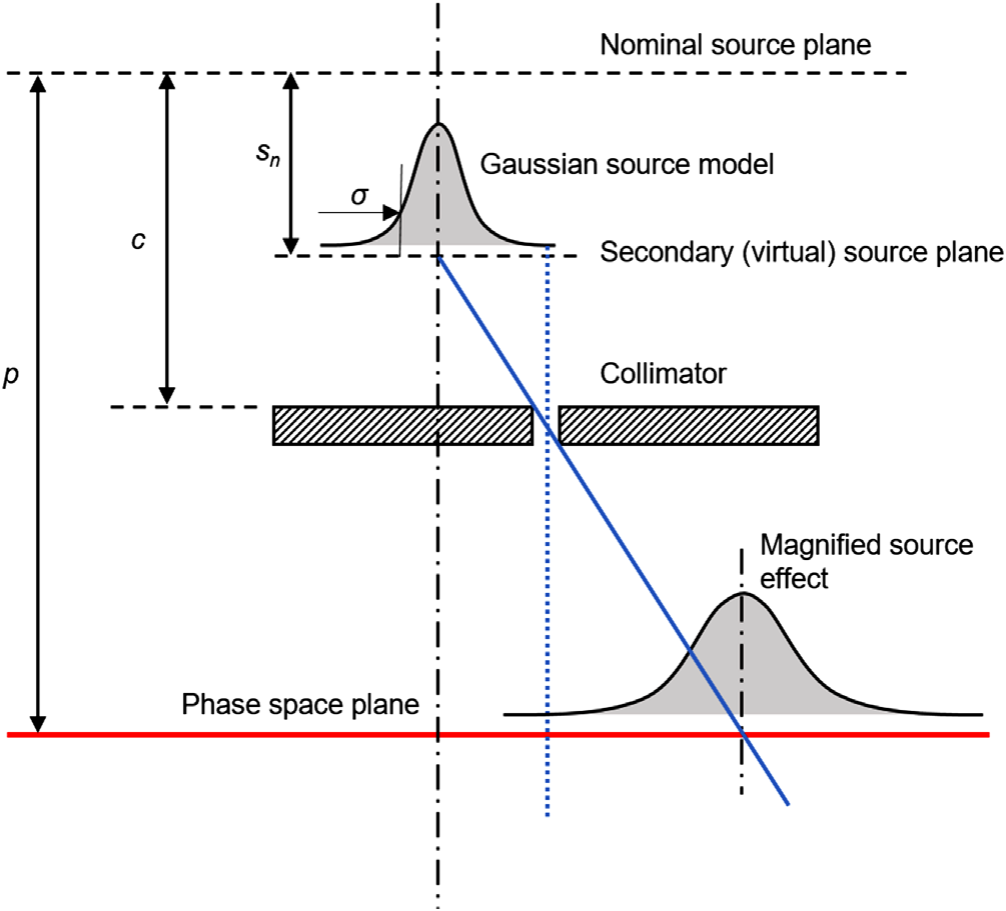
Width of a given source at the phase space plane. The fluence at the phase space plane is equal to the source distribution convolved with the collimator opening. In this instance, the collimator opening forms a delta function, which when convolved with the source distribution, equals the source distribution. The relative positions of source plane, collimator and phase space plane influence the magnification of the source

So far, it was assumed that the sources were point sources. The next step was therefore to introduce the finite source size. Accordingly, taking each source to have a Gaussian profile with a standard deviation of *σ_x_* in the *x*‐direction and *σ_y_* in the *y*‐direction, the width of the source at the phase space was given by the construction in Figure 3:

(8a) $$\sigma_{xp}=\frac{p-c}{c-s_{n}}\;\sigma_{x},$$

(8b) $$\sigma_{yp}=\frac{p-c}{c-s_{n}}\sigma_{y},$$

where *c* was the distance of the collimator from the nominal beam source.

The effect of the finite source size on the fluence distribution at the phase space was then calculated by convolving the source distribution with the fluence calculated in (7):

(9) $${\Phi^{\prime }}_{p}\left(i,j\right)=M\cdot A\cdot F\left(A\right)\cdot C\cdot \Phi_{p}\left(i,j\right)⊗\Omega \left(i,j\right),$$

where Ω(i,j) was a two‐dimensional Gaussian function with standard deviation given in Equation (8). The variable *M* was the number of monitor units specified for the beam in question and *A* was the total open area of the beam aperture, in mm^2^ at the isocenter plane. Note that this could be considered as the integral of $\omega ({x^{\prime }}_{dij},{y^{\prime }}_{dij},{z^{\prime }}_{dij})$ with respect to *dx′* and *dy′*. The area was included to ensure that the appropriate number of particles was transported relative to the required dose and beam aperture.^26^ *F*(*A*) was a collimator scatter factor and *C* was an absolute calibration factor which ensured that the phase space represented a number of particles to be transported. In principle, this convolution step was accomplished by Fourier transforming both the fluence distribution and the Gaussian function, multiplying in Fourier space, and then inverse transforming.

The final step in the calculation of the phase space was to replicate the fluence calculated in Equation (9) and multiply by the energy spectrum, λ(e):

(10) $$\Phi_{\mathrm{pije}}^{\prime }=\Phi_{\mathrm{pij}}^{\prime }\lambda \left(e\right).$$

Thus, for each source, *n*, a collection of particles, $\Psi_{\mathrm{nije}}$, was created, indexed by *n*, *i*, *j* and *e*:

(11) $$\Psi_{\mathrm{nije}}=\left[x_{\mathrm{pij}},y_{\mathrm{pij}},z_{\mathrm{pij}},{\hat{x}}_{\mathrm{pij}},{\hat{y}}_{\mathrm{pij}},{\hat{z}}_{\mathrm{pij}},{\Phi^{\prime }}_{\mathrm{pije}}\right].$$

### 2.2 Numerical implementation

In this work, the phase space consisted of 800 × 800 discrete points, with a spacing at the isocenter plane of 0.5 mm, so as to cover the maximum aperture of the accelerator.

For computational efficiency, the phase space was not actually constructed and stored. Instead, a series of particles were initiated in the Monte Carlo code and rejection sampling was used to select the position of each particle in the phase space grid so that the relative probability was proportional to the fluence distribution $\Phi_{\mathrm{pij}}^{\prime }$ (see Equation (9)). During this process, for each beam in turn, elements of the phase space with an intensity of less than 1% of the maximum intensity for that beam were neglected. Including all phase space elements in the Monte Carlo simulation resulted in the rejection sampling spending an excessive length of time adding particles outside of the beam itself, with a consequent dramatic increase in calculation time. Neglecting the near‐zero elements of the phase space was found to be much more efficient. The energy was further sampled by rejection sampling of the energy spectrum λ(e), thereby satisfying Equation (10), and the rest of the coordinates in Equation (11) were then constructed for that particle.

Table 1 gives the corresponding source‐specific parameters used in this work for flattened beams, while Table 2 gives the parameters for FFF beams. The source position for source 2 was based on the lower edge of the flattening filter although representing scatter from the primary collimator in the case of FFF beams. The source weights and widths in RayStation were the result of carrying out the beam modeling process in RayStation, and represented the standard clinical beam models. These values were used as starting values for the phase space model and in some cases were adequate without further adjustment. In the phase space model, the source weights were manually adjusted to give good agreement with RayStation in the region just outside of the beam aperture for simple beams (see Section 2.4 below). The source widths were also adjusted to give good agreement in the penumbra region. Table 3 gives the source‐independent parameters, based on the geometry of the accelerator.

**TABLE 1:** Source‐specific model parameters for flattened beams

	RayStation		DPM	
Parameter	Source 1	Source 2	Source 1	Source 2
Position relative to nominal source (mm)	0.0	150.0	0.0	150.0
Source weight (relative units)	1.0	0.08	0.94	0.06
Source width (standard deviation) in IEC 61217 *x*‐direction (mm)	0.8	24.0	1.5	24.0
Source width (standard deviation) in IEC 61217 *y*‐direction (mm)	1.0	24.0	1.5	24.0

**TABLE 2:** Source‐specific model parameters for FFF beams

	RayStation		DPM	
Parameter	Source 1	Source 2	Source 1	Source 2
Position relative to nominal source (mm)	0.0	150.0	0.0	150.0
Source weight (relative units)	1.0	0.04	0.96	0.04
Source width (standard deviation) in IEC 61217 *x*‐direction (mm)	0.6	25.0	2.0	24.0
Source width (standard deviation) in IEC 61217 *y*‐direction (mm)	0.3	25.0	0.5	24.0

**TABLE 3:** Source‐independent model parameters

Parameter	Value
Phase space width in IEC 61217 *x*‐direction (pixels)	800
Phase space width in IEC 61217 *y*‐direction (pixels)	800
Phase space resolution in IEC 61217 *x*‐direction (mm at isocenter)	0.5
Phase space resolution in IEC 61217 *y*‐direction (mm at isocenter)	0.5
Phase space edge position in IEC 61217 *x*‐direction (mm at isocenter)	−200.0
Phase space edge position in IEC 61217 *y*‐direction (mm at isocenter)	−200.0
Phase space position relative to nominal source (mm)	548.0^a^
Collimator position relative to nominal source (mm)	401.8^b^

^a^ Versa HD accessory ring.

^b^ Versa HD multileaf collimator bottom of leaves.

The primary fluence profile, $\varphi ({r^{\prime }}_{dij})$, was taken directly from the RayStation model without adjustment (Table 4 for flattened beams and Table 5 for FFF beams). The energy spectrum, λ(e), was adjusted uniformly along its energy axis to increase the relative content of high‐energy photons, so that the depth dose for a 100 mm × 100 mm beam was correct (Table 6 for both flattened and FFF beams). To ensure the correct absolute dose, the collimator scatter factor, *F*(*A*), was set to unity for a 100 mm × 100 mm beam and the calibration factor *C* was then calculated by adjusting so that the beam dose agreed with RayStation. This approach, based on the 100 mm × 100 mm beam, mirrored that used in RayStation and other treatment planning systems, as well as reflecting the practical definition of monitor units on the linear accelerator. The value used was 2.02 × 10^–14^ for flattened beams and 2.18 × 10^–14^ for FFF beams. The collimator scatter factors, *F*(*A*), were then determined for the other field sizes by initially setting all values to unity and then adjusting so that the outputs of the square beams were correct in relation to the corresponding RayStation beams (Table 7).

**TABLE 4:** Primary fluence profile used for the generation of the phase space for flattened beams

Off‐axis position (mm)	Relative intensity
0.0	1.000
10.0	1.003
20.0	1.006
50.0	1.020
70.0	1.025
90.0	1.030
100.0	1.035
150.0	1.047
175.0	1.051
190.0	1.055
200.0	1.060
210.0	1.060
230.0	1.000
260.0	0.500
261.0	0.000
500.0	0.000

**TABLE 5:** Primary fluence profile used for the generation of the phase space for FFF beams

Off‐axis position (mm)	Relative intensity
0	1.000
20	0.971
50	0.865
70	0.787
90	0.720
100	0.684
150	0.552
175	0.499
190	0.475
200	0.455
210	0.435
225	0.410
240	0.375
250	0.345
255	0.325
258	0.000

**TABLE 6:** Energy spectrum used for the generation of the phase space

Energy (MeV)	Relative intensity (flattened beams)	Relative intensity (FFF beams)
0.50	0.04184	0.08990
1.00	0.07318	0.09820
1.50	0.08604	0.06197
2.00	0.07853	0.05149
2.50	0.06149	0.04309
3.00	0.05403	0.03776
3.50	0.03800	0.03369
4.00	0.02962	0.03032
5.00	0.02559	0.02645
6.00	0.01542	0.02408

**TABLE 7:** Collimator scatter factors for flattened and FFF beams

Field size (mm)	Scatter factor (flattened beams)	Scatter factor (FFF beams)
10.0	0.970	0.980
20.0	0.930	0.980
30.0	0.940	0.960
50.0	0.975	0.985
100.0	1.000	1.000
150.0	1.015	1.000
200.0	1.030	1.015
400.0	1.040	1.020

### 2.3 Coupling with Monte Carlo dose calculation

The phase space model was applied to the DPM Monte Carlo code.^24^ This was originally designed for simulation of electron beams and subsequently extended to the handling of photon beams. It used a mixed scheme to model particle interactions, with large energy‐loss interactions being handled in an analogue fashion, and small energy‐loss interactions being approximated by the continuous slowing‐down approximation. By reformulating the Goudsmit–Saunderson multiple‐scattering theory^27^, ^28^, ^29^ to be independent of calculation step size, the facility to compute dose using longer step sizes, while maintaining the accuracy of the modeling, was provided. These longer step sizes, including across tissue heterogeneities, allowed for much faster calculation of the dose distribution, and hence potential clinical application.

The implementation of this code used in the present study was written in C++ and was designed to take advantage of modern multi‐core central processing units (CPUs).^30^ It was run on a 4‐core CPU with eight threads running at 3.4 GHz. Tissue type was determined using a stoichiometric calibration,^31^ in which a conversion table of Hounsfield number to relative electron density was used to determine relative electron density. An empirical conversion formula was then used to convert relative electron density into physical density, and a series of discrete ranges of physical density were then defined, each corresponding to a different tissue type, with tabulated properties.^32^

The program read the IMRT plan from a DICOM file, computed the phase space from the plan, and then applied the phase space to the Monte Carlo simulation. The requested statistical uncertainty was 1.5%, following Goodall and Ebert.^33^ The final dose distribution represented the dose due to the arbitrary number of particles required to give the requested statistical uncertainty, and was therefore unrelated to the number of monitor units in the plan. The dose distribution was therefore scaled by ∑ijΦ′pij/∑ijΦ′pijHH, where $\sum_{ij}{\Phi^{\prime }}_{pij}$ (see Equation (10)) represented the integral fluence and *H* was the total number of particles transported. The denominator of this factor effectively converted the dose distribution into dose per particle and the numerator then multiplied it by the exact number of particles calibrated according to the monitor units. A median window filter with a radius of three voxels was applied to the final dose distribution to reduce the statistical noise.^34^, ^35^ The method computed dose to medium in medium.

### 2.4 Application to simple beams

To test the accuracy of the phase space implementation and subsequent Monte Carlo algorithm, the dose distribution in a homogeneous water phantom of dimensions 300 mm width (A‐B direction) × 300 mm height × 300 length (superior‐inferior direction) was calculated for square fields of width 10, 20, 30, 50, 100, 150 and 200 mm. Off‐axis fields were also considered, consisting of square fields of width 30 and 50 mm, with the center of the field located either 50 or 100 mm to the +*x* and +*y* direction in the beam's eye view (IEC61217 convention). The resolution of the phase space was 0.5 mm × 0.5 mm and the dose grid resolution was 2.0 mm × 2.0 mm × 2.0 mm, which represented a typical resolution in a clinical setting. For the field of width 10 mm, the median window filter was reduced to a width of one voxel to avoid excessively smoothing the already small high‐dose region. Dose to medium in medium was computed. The resulting dose distributions were exported from the DPM software as a DICOM‐RT dose object and then imported into RayStation, where the dose was compared with that computed using RayStation's own collapsed cone convolution algorithm on an identical grid resolution. The collapsed cone convolution algorithm was used in contrast to RayStation's Monte Carlo photon algorithm for two reasons: (a) the phase space parameters were taken from the convolution model so the convolution model was the logical selection for comparison, and (b) to avoid adding statistical uncertainties from two Monte Carlo results.

Both the DPM dose and the RayStation dose were also exported to Verisoft (v8.0, PTW, Freiburg, Germany). Output factors, calculated as the dose at the center of the field at 100 mm depth in the phantom, relative to the dose at the center of a 100 mm × 100 mm field at the same depth, were computed. Gamma statistics were also computed for 2% of 100 cGy and 2 mm. The percentage of dose voxels with a gamma of less than unity was recorded, considering those voxels with a dose higher than 10% of the maximum RayStation dose.

### 2.5 Application to IMRT plans

The method was then applied to two stereotactic ablative body radiotherapy (SABR) treatment plans: a prostate and a lung plan. These plans were used at this center as part of a multi‐institutional study of real‐time adaptive radiotherapy,^36^ so the validity and accuracy of the plans were well understood. (The plans were produced using Pinnacle^3^ v9.10 (Philips Radiation Oncology Systems, Madison, WI) but recalculated in RayStation for the purposes of this dose comparison study.) The prostate clinical target volume (CTV) was 55.7 cm^3^ and the contouring was according to RTOG 0938.^37^ The margin between the CTV and the planning target volume (PTV) was 3 mm posteriorly and 5 mm elsewhere. The treatment plan consisted of seven equally spaced coplanar beams, with a total of 28 segments, for step‐and‐shoot delivery with the 6 MV beam of a Versa HD accelerator. Both flattened and FFF versions of the plan were available for comparison.

The gross tumor volume (GTV) of the phase I non‐small cell lung cancer patient was 7.7 cm^3^ and the CTV was taken to be equal to the GTV. The PTV margin was 5 mm in all directions. No internal target volume was defined as the treatment plan was designed to be used in conjunction with multileaf collimator tracking.^36^ The treatment plan consisted of 15 equally spaced coplanar beams, with a total of 30 segments for step‐and‐shoot delivery. Both flattened and FFF versions of the treatment plan were considered.

All of these plans were recalculated using the phase space model and DPM code, as well as in RayStation using collapsed cone convolution. The resolution of the phase space was 0.5 mm × 0.5 mm in DPM and the dose grid resolution was 2.0 mm × 2.0 mm × 2.0 mm in both DPM and RayStation, in accord with normal clinical practice for these SABR treatment plans. Note that DPM calculated dose to medium in medium, whereas RayStation calculated dose to water of modified density.

Verisoft was also used to compute gamma statistics for the DPM and RayStation doses, for 2% of the prescribed dose and 2 mm. Note that the plans were stereotactic, so the maximum dose was considerably higher than the prescribed dose. The percentage of dose voxels with a gamma of less than unity was recorded, considering those voxels with a dose higher than 10% of the maximum RayStation dose.

## 3 RESULTS

### 3.1 Phase space

**FIGURE 4:**
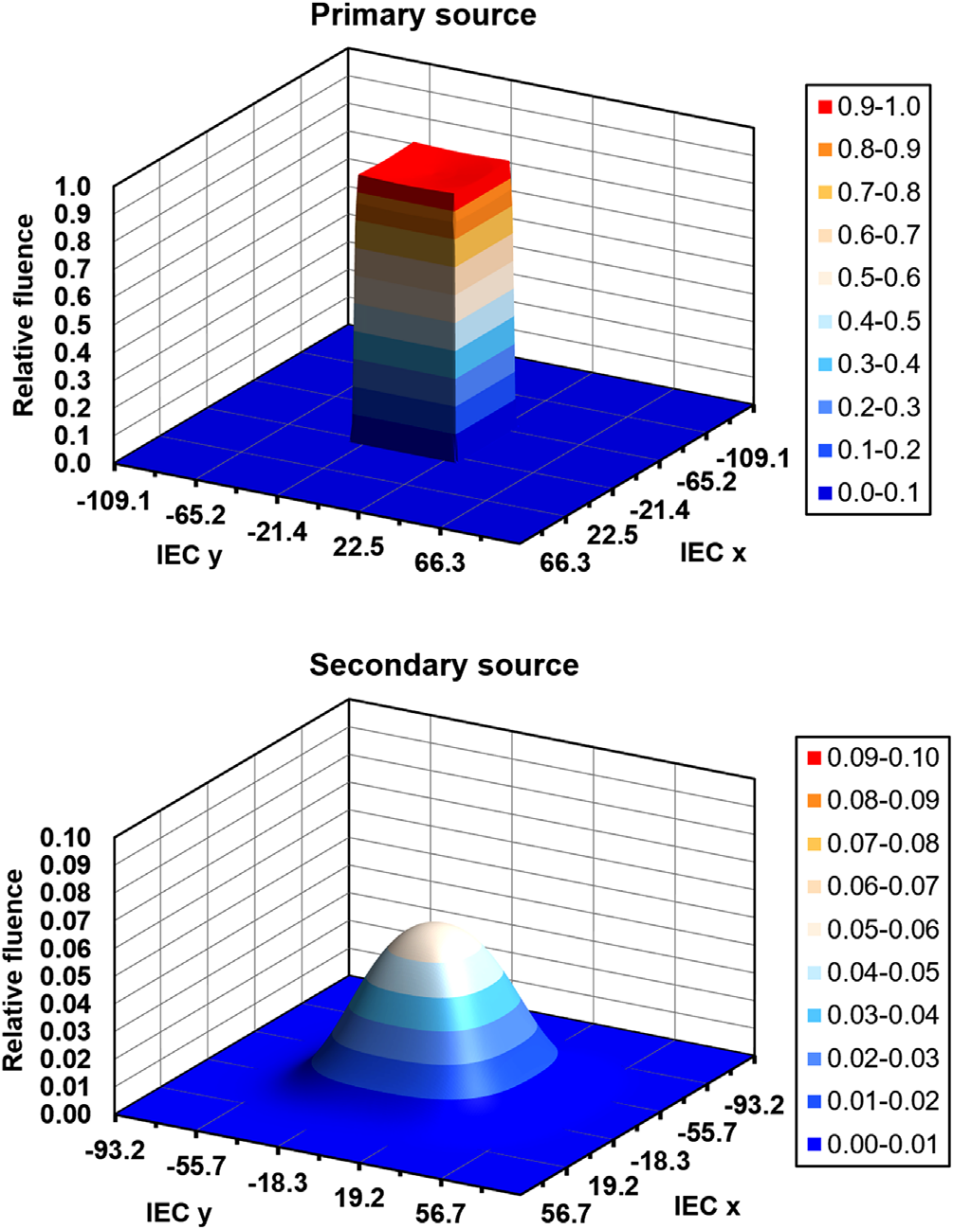
Fluence distribution of the phase space with a reduced resolution of 2 mm × 2 mm (specified at the isocenter) and a single photon energy of 1 MeV for a 100 mm × 100 mm flattened beam. Note that the horizontal and vertical axes have different scales in the two parts of the figure, the former due to the different divergence of the primary and secondary sources, and the latter due to the different magnitudes of the sources

A version of the phase space with reduced spatial resolution and with a single photon energy is shown in Figure 4 for a 100 mm × 100 mm flattened beam. The primary source is of the order of 1 mm so the blurring due to the source size is minimal. The result is that the fluence closely follows the shape of the aperture, with magnitude largely governed by the supplied radial fluence profile. In contrast, the secondary source is broad (24 mm standard deviation), so the fluence is dominated by Gaussian blurring.

### 3.2 Application to simple beams

Numbers of particle histories to give the required statistical uncertainty of 1.5% for a sample of cases are shown in Table 8. Number of histories, and hence dose calculation time is approximately proportional to the total area of the beam aperture, but also depends on the volume of the high‐dose region over which statistical uncertainty is measured. For single beams, the high‐dose region is somewhat extended, so the calculation takes 5 min for a 100 mm × 100 mm square field on the 4‐core CPU used in this work and correspondingly shorter or longer for the smaller and larger field sizes.

**TABLE 8:** Number of photon histories required for calculation with flattened and FFF beams

Field size/Treatment plan	Histories (flattened beams)	Histories (FFF beams)
10.0 mm	3.50 × 10^6^	3.70 × 10^6^
30.0 mm	2.90 × 10^7^	3.00 × 10^7^
50.0 mm	7.98 × 10^7^	8.14 × 10^7^
100.0 mm	3.13 × 10^8^	3.10 × 10^8^
200.0 mm	1.22 × 10^9^	1.10 × 10^9^
Prostate IMRT	3.28 × 10^7^	3.55 × 10^7^
Lung IMRT	6.63 × 10^7^	7.53 × 10^7^

**FIGURE 5:**
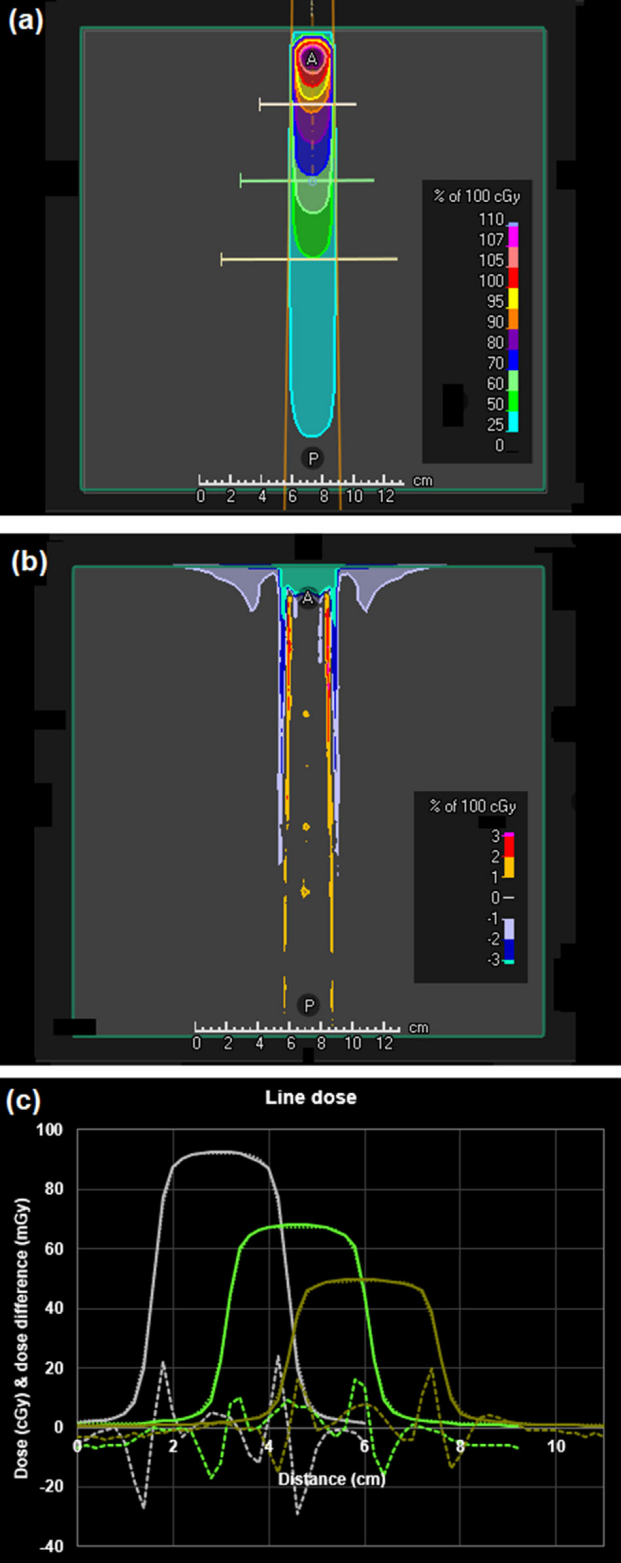
(a) Dose distribution calculated by the phase space model and DPM for a 30 mm × 30 mm square flattened beam. (b) Dose difference map of DPM in relation to RayStation convolution calculation. (c) Dose profiles through the central axis of the beam at a depth of (left) 50 mm, (center) 100 mm and (right) 150 mm (as indicated by the lines in (a)). Solid line: DPM (cGy); dotted line: RayStation convolution (cGy); dashed line: difference (mGy)

**FIGURE 6:**
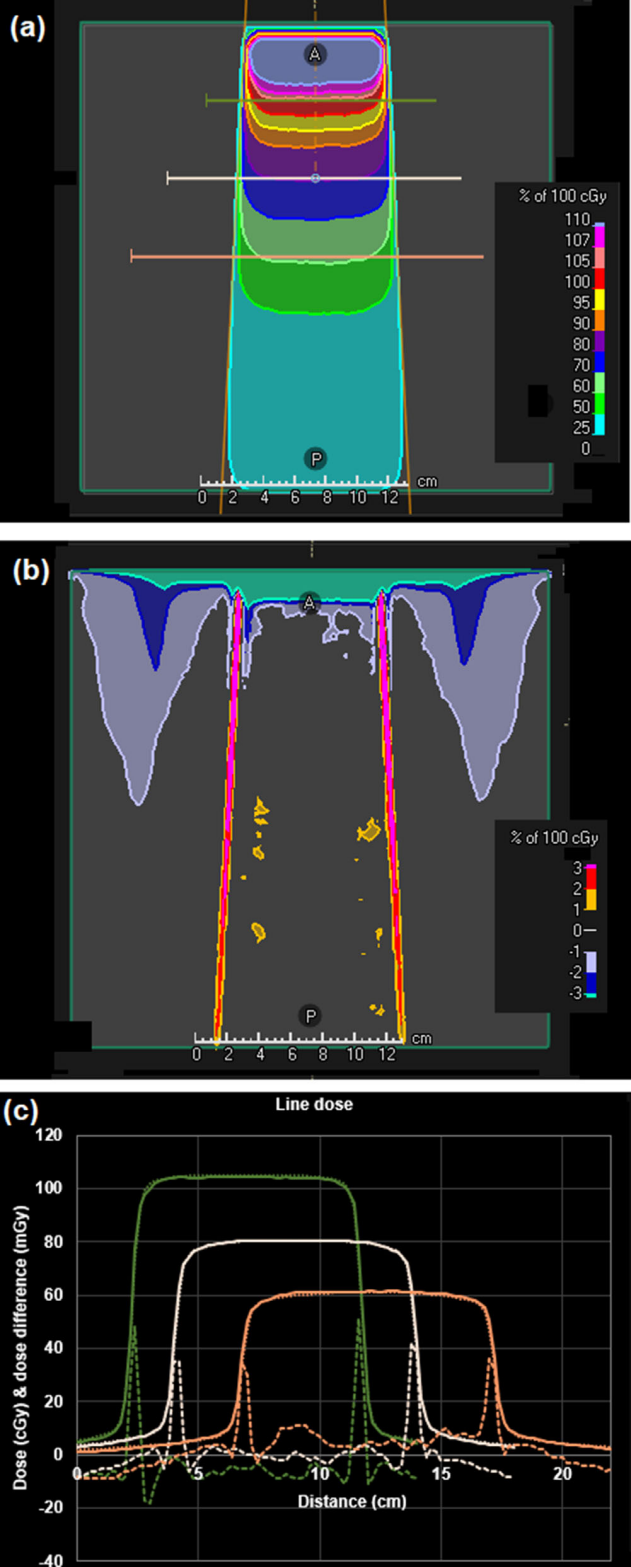
(a) Dose distribution calculated by the phase space model and DPM for a 100 mm × 100 mm square flattened beam. (b) Dose difference map of DPM in relation to RayStation convolution calculation. (c) Dose profiles through the central axis of the beam at a depth of (left) 50 mm, (center) 100 mm and (right) 150 mm (as indicated by the lines in (a)). Solid line: DPM (cGy); dotted line: RayStation convolution (cGy); dashed line: difference (mGy)

**FIGURE 7:**
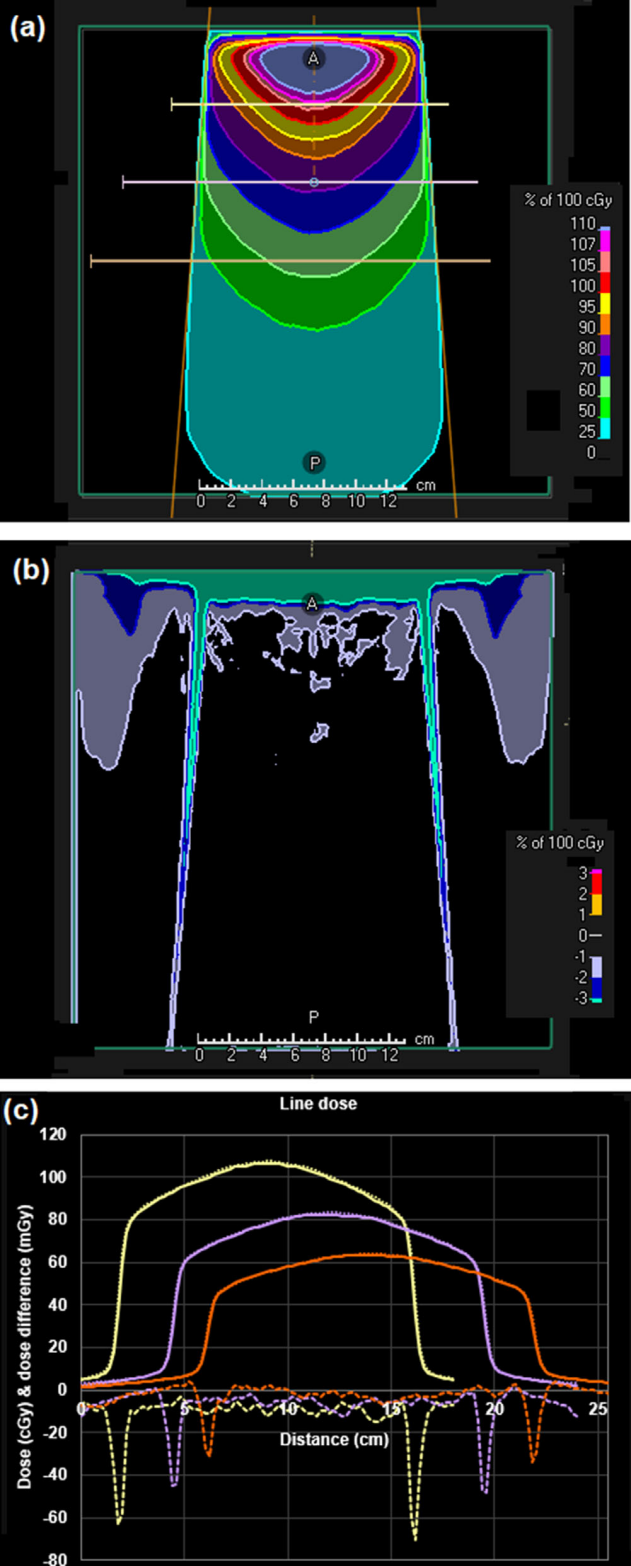
(a) Dose distribution calculated by the phase space model and DPM for a 150 mm × 150 mm square FFF beam. (b) Dose difference map of DPM in relation to RayStation convolution calculation. (c) Dose profiles through the central axis of the beam at a depth of (left) 50 mm, (center) 100 mm and (right) 150 mm (as indicated by the lines in (a)). Solid line: DPM (cGy); dotted line: RayStation convolution (cGy); dashed line: difference (mGy)

**FIGURE 8:**
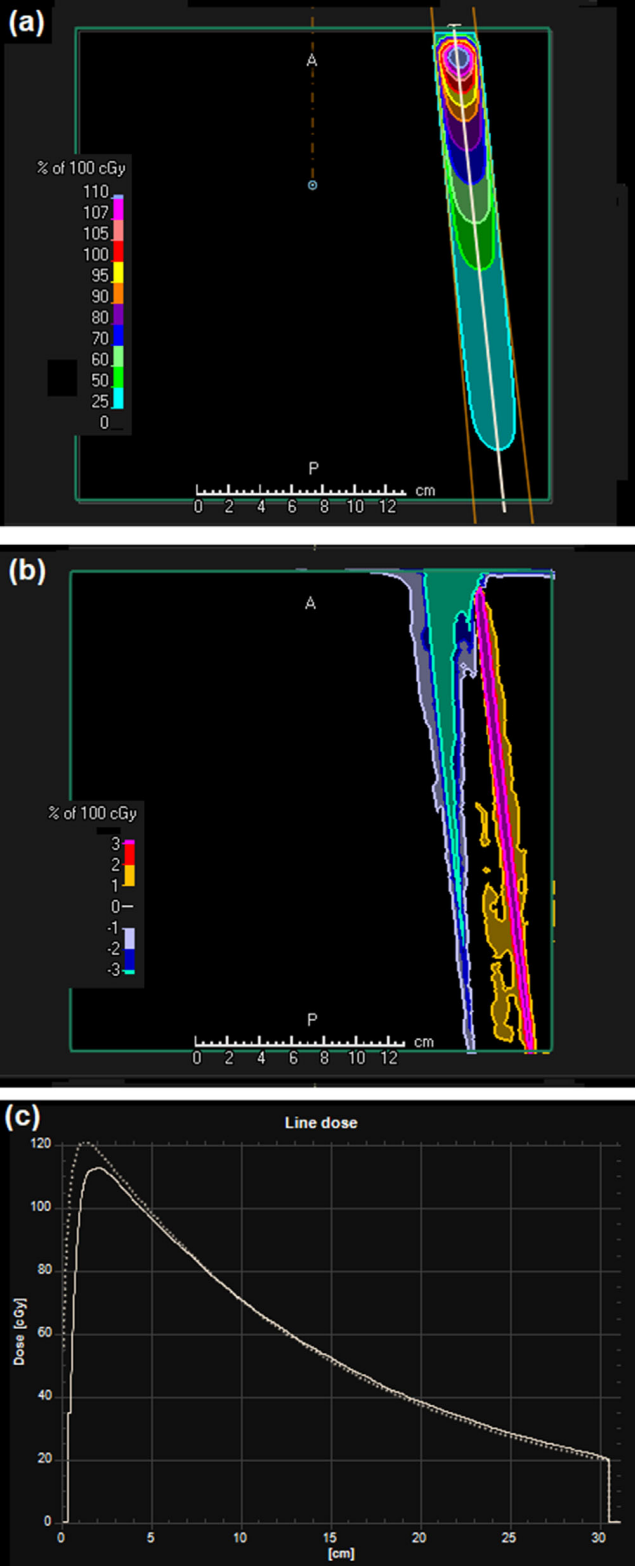
(a) Dose distribution calculated by the phase space model and DPM for a 30 mm × 30 mm square beam, 100 mm off‐axis toward +*x* (IEC61217 convention). (b) Dose difference map of DPM in relation to RayStation convolution calculation. (c) Oblique depth‐dose through the center of the beam (as indicated by the line in (a)). Solid line: DPM; dotted line: RayStation convolution

A difference map between the DPM dose distribution and the RayStation convolution dose distribution is shown in Figure 5 for a 30 mm × 30 mm flattened beam. Similar results are obtained for fields down to 10 mm × 10 mm in size. A difference map is shown in Figure 6 for a 100 mm × 100 mm flattened beam. The dose differences are generally less than 2%. The area of larger difference in the buildup region is attributed to the lack of an electron contamination component in the DPM code used for this study. Note that there is an area outside of the beam with a dose difference of 1%–2%. This is due to a small out‐of‐field underestimation of dose by the phase space model, exacerbated by the lack of electron contamination in the DPM calculation. The effect is not seen further laterally or at deeper depths. Figure 7 shows the results for a 150 mm × 150 mm FFF beam. The dose agreement between DPM and RayStation is generally better than 1%, with the exception of the regions of high dose gradient, and the region outside of the beam superficially, the latter being in the order of 2%, diminishing to zero at greater depths. An example of an off‐axis field is shown in Figure 8. The agreement of dose in the penumbra region is not quite as uniform as with symmetric fields, but still in good agreement. The depth dose is also in reasonable agreement, except superficially, where the absence of electron contamination in the Monte Carlo result is evident.

The output factors are shown in Table 9, where it can be seen that the agreement between DPM and RayStation is generally within ±1%. The gamma agreement is shown in Table 10. The majority of doses for DPM are within 2% and 2 mm of the corresponding RayStation doses. However, some allowance needs to be made for the lack of electron contamination in the Monte Carlo results, which reduces the gamma pass rate by up to approximately 10%, with greater impact for small fields, where the differences in the buildup region account for a relatively large proportion of points evaluated.

**TABLE 9:** Output factors for simple beams

Field offset	Field width (mm)	DPM (flattened beams)	RayStation (flattened beams)	DPM (FFF beams)	RayStation (FFF beams)
None	10	0.690	0.682	0.699	0.703
None	20	0.803	0.796	0.836	0.830
None	30	0.845	0.837	0.885	0.878
None	50	0.900	0.903	0.932	0.924
None	100	1.000	1.000	1.000	1.000
None	150	1.053	1.060	1.033	1.037
None	200	1.086	1.098	1.068	1.061
* X * 50 mm^a^	30	0.851	0.853	0.764	0.761
* X * 50 mm	50	0.914	0.920	0.813	0.803
* X * 100 mm	30	0.862	0.862	0.608	0.601
X 100 mm	50	0.923	0.933	0.643	0.637
Y 50 mm	30	0.854	0.853	0.764	0.761
Y 50 mm	50	0.911	0.920	0.810	0.803
Y 100 mm	30	0.860	0.863	0.608	0.602
Y 100 mm	50	0.922	0.933	0.639	0.637

^a^ X‐ and Y‐ offset refer to the IEC61217 collimator convention.

**TABLE 10:** Gamma pass rate for simple beams

Field offset	Field width (mm)	Gamma^a^ (flattened beams)	Gamma^a^ (FFF beams)
None	10	97.2	97.5
None	20	77.1	72.5
None	30	85.3	80.9
None	50	90.6	90.2
None	100	93.4	93.9
None	150	92.9	94.3
None	200	76.3	94.9
X 50 mm^b^	30	85.4	83.9
X 50 mm	50	89.1	91.2
X 100 mm	30	80.1	87.1
X 100 mm	50	79.7	91.4
Y 50 mm	30	85.9	83.6
Y 50 mm	50	89.6	91.1
Y 100 mm	30	81.3	85.6
Y 100 mm	50	79.2	90.0

^a^ 2 cGy/2 mm with threshold 10% of maximum dose.

^b^ X‐ and Y‐ offset refer to the IEC61217 collimator convention.

### 3.3 Application to IMRT plans

**FIGURE 9:**
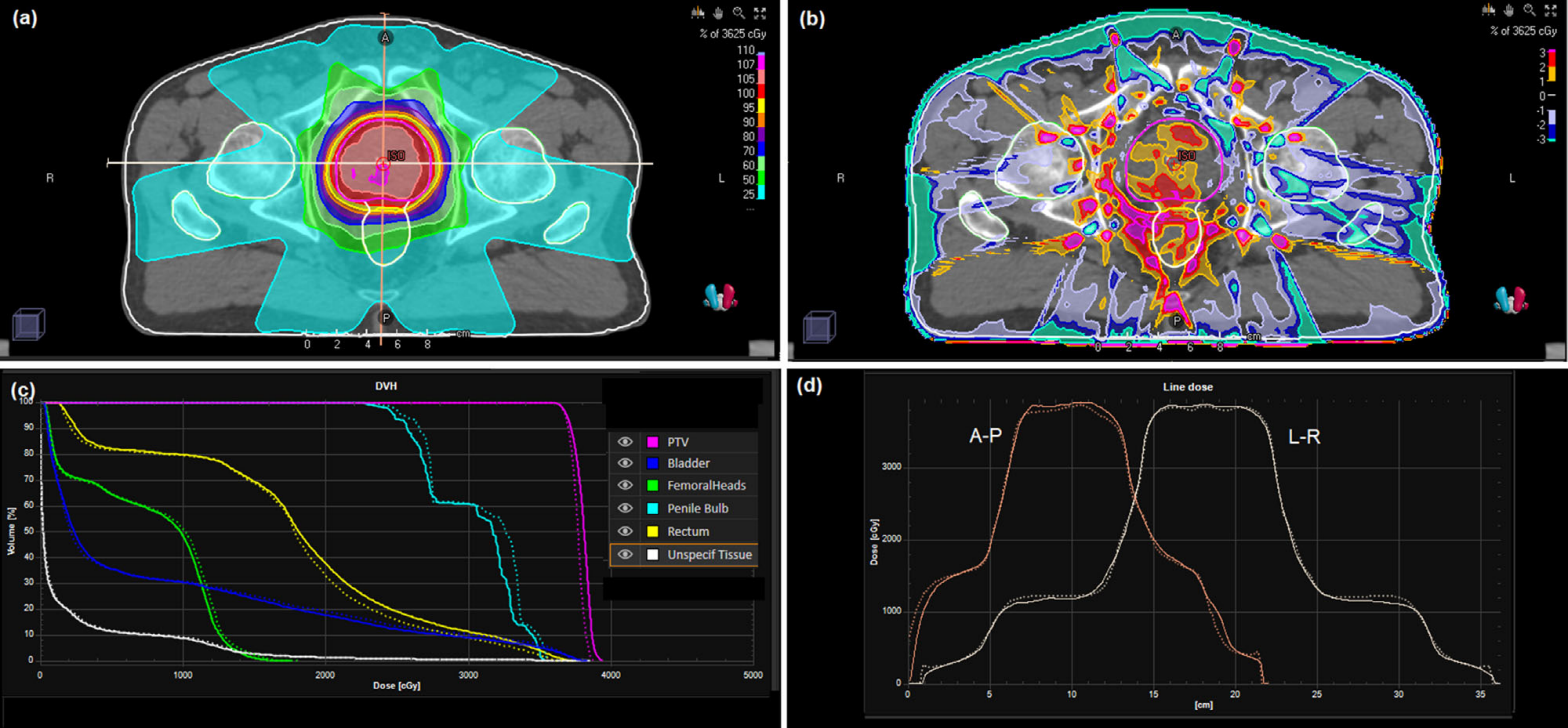
(a) Dose distribution for the prostate IMRT case with flattened beams calculated by the phase space model and DPM. (b) Dose difference map of DPM in relation to RayStation convolution calculation. (c) Dose‐volume histograms for DPM and RayStation convolution. (d) Dose profiles from patient's right to patient's left and from anterior to posterior through the isocenter (as indicated by the lines in (a)). Solid lines: DPM; dotted lines: RayStation convolution

**FIGURE 10:**
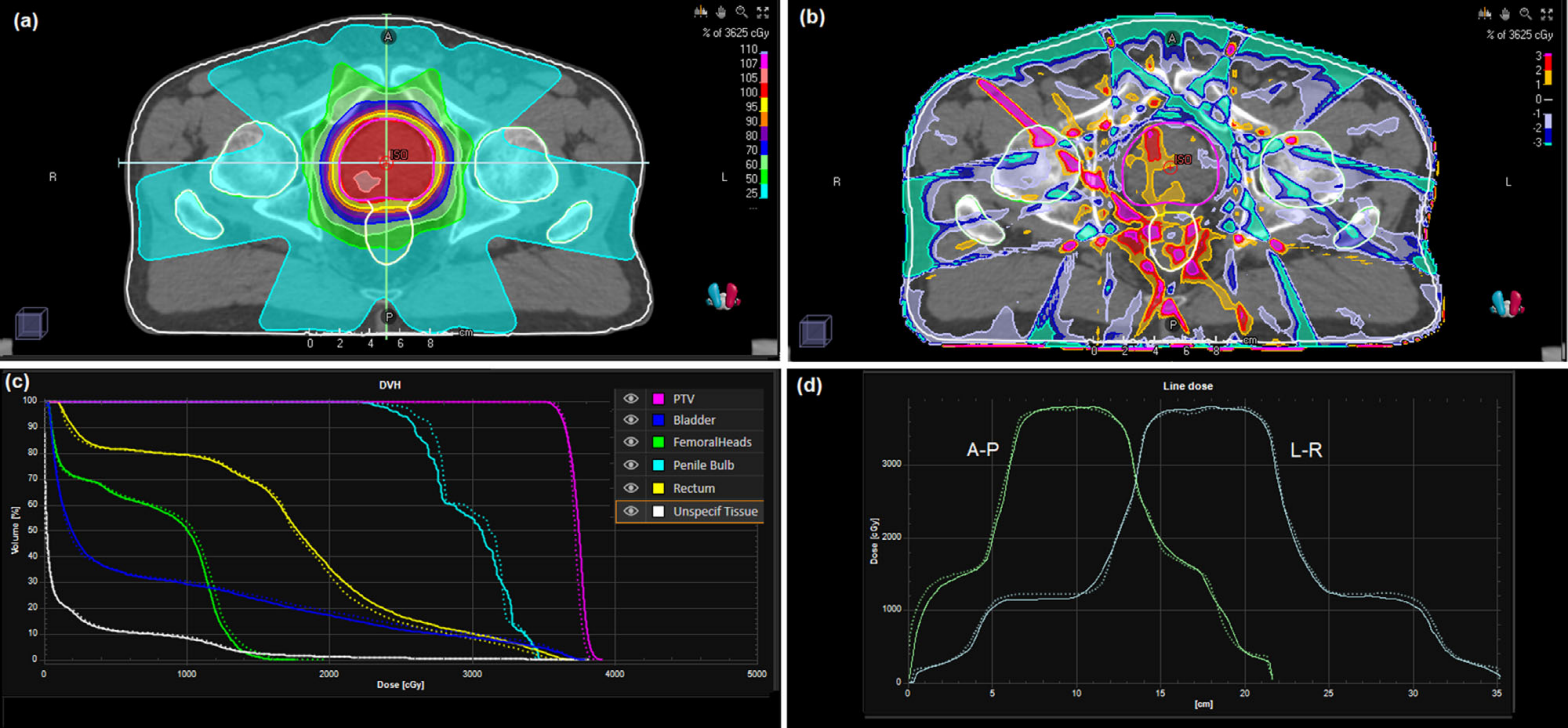
(a) Dose distribution for the prostate IMRT case with FFF beams calculated by the phase space model and DPM. (b) Dose difference map of DPM in relation to RayStation convolution calculation. (c) Dose‐volume histograms for DPM and RayStation convolution. (d) Dose profiles from patient's right to patient's left and from anterior to posterior through the isocenter (as indicated by the lines in (a)). Solid lines: DPM; dotted lines: RayStation convolution

For the complete prostate and lung IMRT plans, the dose calculation takes 3 min. Difference maps between the DPM dose distribution and the RayStation convolution dose distribution are shown in Figure 9 for the prostate case using flattened beams. In general, the dose difference between the two calculation methods is less than ±2%, but the difference increases to ±4% in the regions representing high dose gradients of individual segments. A small degree of smoothing is visible in the dose distribution, due to the filtration used to reduce the statistical noise in the Monte Carlo simulation. The dose‐volume histograms are in good agreement between calculation methods, with the largest differences seen for the penile bulb, which has a small volume and is located very close to the PTV. For the rectum, the difference between calculation methods is greater at higher doses, due to the presence of higher dose gradients at those higher doses. Similar results are also seen for the case of FFF beams (Figure 10). For the rectum, the difference between DPM and RayStation is again greater at higher doses, due to higher dose gradients.

**FIGURE 11:**
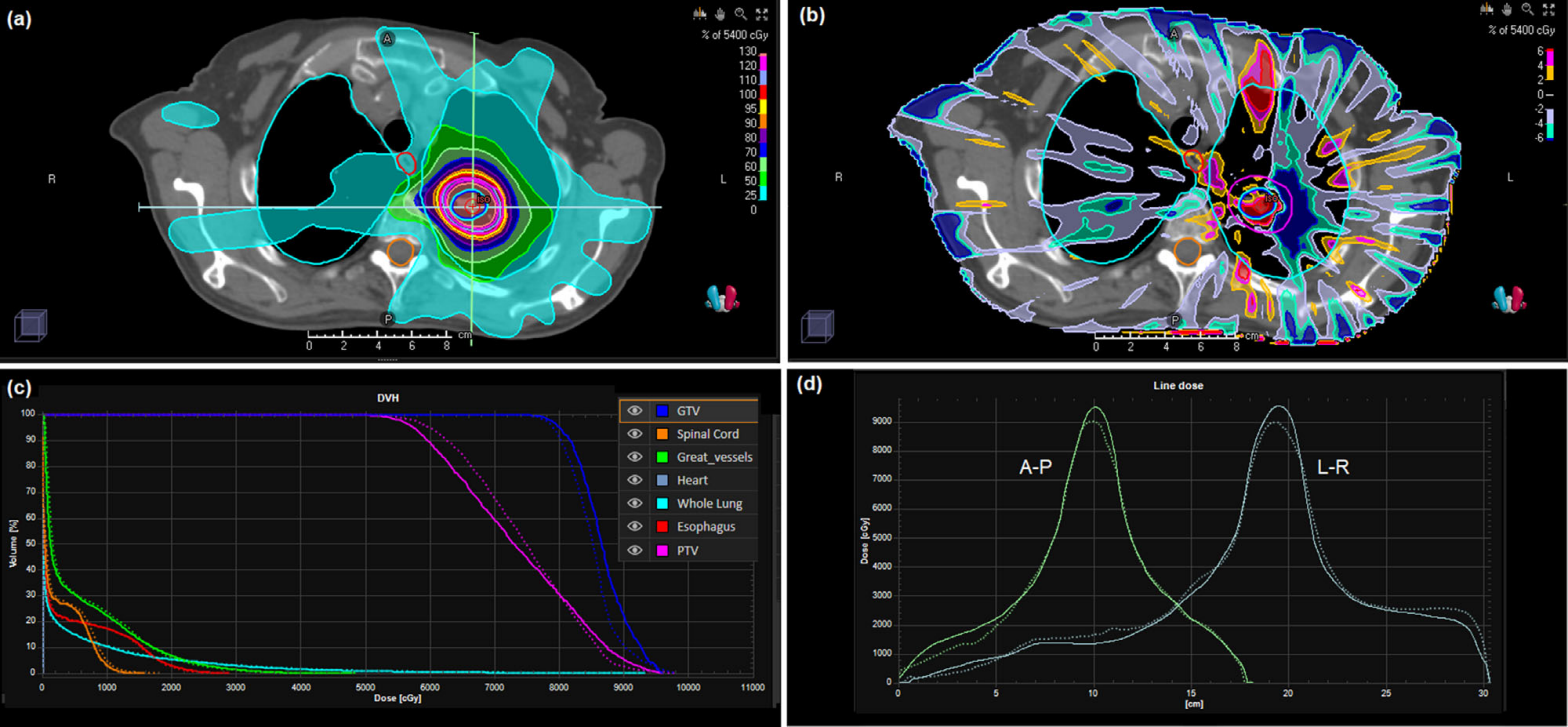
(a) Dose distribution for the lung IMRT case with flattened beams calculated by the phase space model and DPM. (b) Dose difference map of DPM in relation to RayStation convolution calculation. (c) Dose‐volume histograms for DPM and RayStation convolution. (d) Dose profiles from patient's right to patient's left and from anterior to posterior through the isocenter (as indicated by the lines in (a)). Solid lines: DPM; dotted lines: RayStation convolution

**FIGURE 12:**
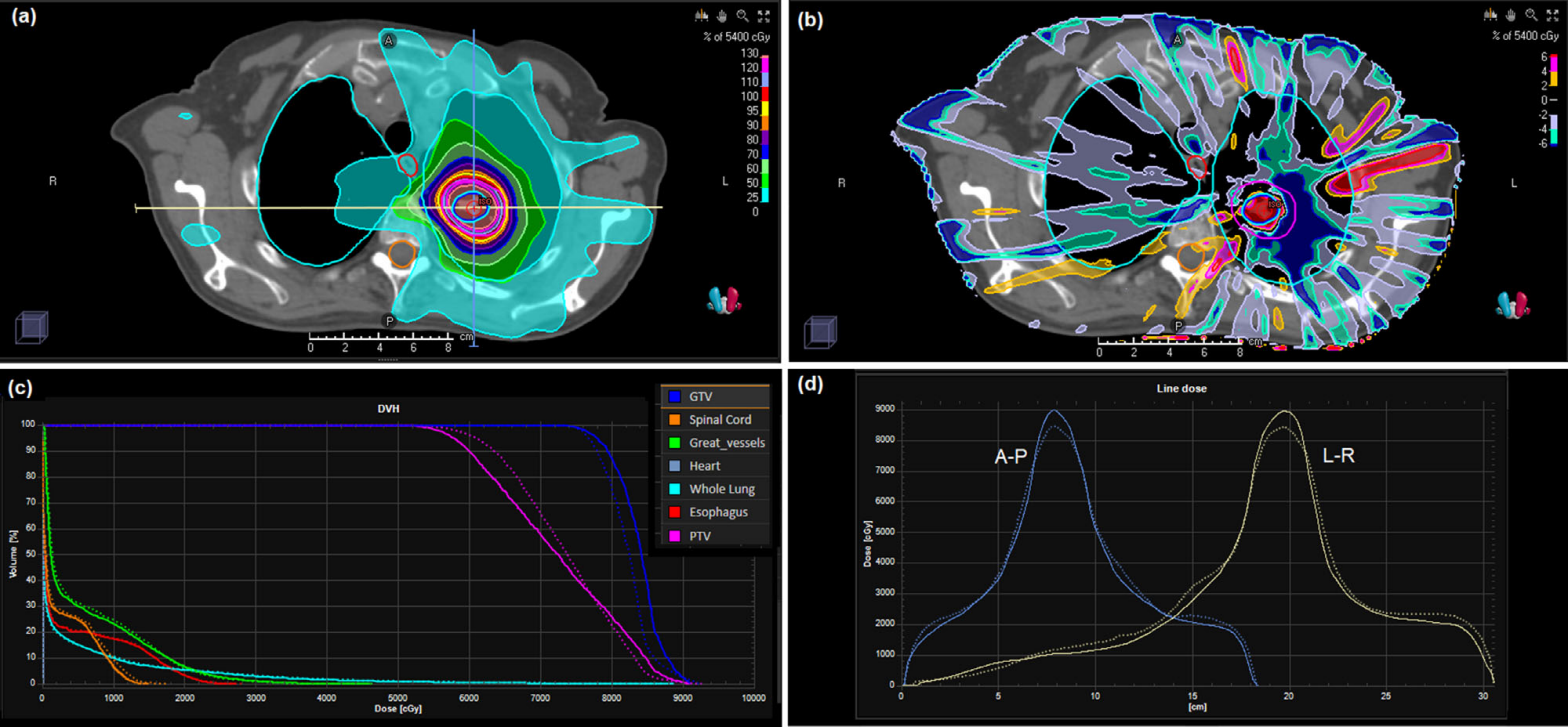
(a) Dose distribution for the lung IMRT case with FFF beams calculated by the phase space model and DPM. (b) Dose difference map of DPM in relation to RayStation convolution calculation. (c) Dose‐volume histograms for DPM and RayStation convolution. (d) Dose profiles from patient's right to patient's left and from anterior to posterior through the isocenter (as indicated by the lines in (a)). Solid lines: DPM; dotted lines: RayStation convolution

The results are shown in Figures 11 and 12 for the lung case with flattened and FFF beams respectively. The largest difference in the dose distributions is seen centrally within the PTV, in the order of 6%, with the edges of the PTV exhibiting better dosimetric agreement. The dose profiles show that the dose fall‐off around the PTV is in good agreement, but that there are some differences between the two calculation methods in the beam penumbra further away from the target volume. The dose‐volume histograms for the normal tissues are in good agreement between convolution and Monte Carlo calculations.

Gamma results for all of these plans are summarized in Table 11. Broadly, the gamma results reflect the reasonable agreement of the Monte Carlo and convolution algorithms. However, there are some differences between the algorithms, including the absence of electron contamination in the incident beams in the case of DPM, which lowers the percentage of dose voxels with gamma less than unity.

**TABLE 11:** Gamma pass rate for the IMRT plans

Treatment plan	Gamma tolerance^a^	Gamma^b^ (flattened beams)	Gamma^b^ (FFF beams)
Prostate IMRT	2%/2 mm	79.2	80.6
Prostate IMRT	3%/3 mm	91.3	93.1
Lung IMRT	2%/2 mm	66.2	63.2
Lung IMRT	3%/3 mm	84.3	83.4

^a^ Percentage refers to percentage of prescribed dose.

^b^ Threshold 10% of maximum dose in the RayStation plan.

## 4 DISCUSSION

An accurate phase space model is essential for reliable dose calculation using Monte Carlo simulation. The final accuracy of the calculation depends on both the accuracy of the phase space and the accuracy of the Monte Carlo simulation in the patient, so even if the Monte Carlo algorithm itself is highly accurate, the final results are not accurate if the phase space is unreliable. Although it is difficult to estimate the sources of uncertainty accurately, the results in this study indicate that the standard deviation of uncertainty in the phase space model is around 1% and the statistical uncertainty in the Monte Carlo calculation is around 1.5%. These uncertainties combine in quadrature, and observations may be up to two standard deviations from the mean. In the lung case particularly, there are also differences between convolution/superposition and Monte Carlo simulation due to different modeling of the physical processes involved in dose deposition. In this situation, the Monte Carlo result is likely to be more accurate due to the more comprehensive modeling of particle scatter in the inhomogeneous media. Monte Carlo simulation is considered to be the gold standard for dose calculation, which is the motivation for using it in the real‐time adaptive context, and the lung IMRT case demonstrates the improvement in accuracy. The difference between the two dose calculation methods has a standard deviation in the order of 2%.

In addition, the generation of the phase space must be fast for clinical application, particularly in the context of real‐time adaptive radiotherapy. The method presented offers a method for generation of a phase space which is both efficient to calculate and suitably accurate. It therefore opens up scope for Monte Carlo simulation in a real‐time context.

For practical purposes, it is also helpful if the phase space model is compatible with a commercial treatment planning system. In this case, the phase space is chosen to agree as closely as possible with the deterministic dose calculation algorithm on the RayStation treatment planning system. The RayStation treatment planning system also provides a Monte Carlo algorithm, but this is not used for the present study as the parameters of the convolution model are used for the phase space, so the convolution calculation is the natural choice of dose algorithm for comparison. This also avoids the buildup of statistical uncertainty due to the comparison of two Monte Carlo algorithms. The collapsed cone convolution/superposition algorithm used by RayStation is also the standard clinical algorithm used at this center, so it is the natural choice for comparison.

The doses calculated by DPM for simple beams in a water‐equivalent phantom show good agreement with the RayStation doses. The largest differences occur in the buildup region, because the Monte Carlo phase space does not include electrons, so the Monte Carlo results show lower dose in that region. This is also reflected in the gamma results. There is also reasonable agreement between the Monte Carlo and convolution methods for the prostate case, although again affected by the differences in the buildup region. Some larger differences are apparent in the lung case, but perfect agreement is not expected in this plan due to the nature of calculating dose in a very inhomogeneous environment using convolution‐superposition and Monte Carlo methods. In particular, the two calculations account for loss of lateral electronic equilibrium in very different ways.

Some difference between RayStation and DPM is expected for both of the patient cases due to the calculation of absorbed dose to water of modified density in RayStation and absorbed dose to medium in DPM. However, the difference in dose in this scenario is shown by Ma and Li to be much less than when comparing absorbed dose to water of modified density with absorbed dose to water in medium.^38^ The difference between absorbed dose to water of modified density and absorbed dose to medium is also greatest for high‐density media such as bone, so is not considered to have much impact on the dose comparison in the target regions of either of the clinical plans chosen in this study. The reader is referred to the work of Ma and Li for a full discussion, with various simulations, on this subject.^38^ In general, it is recognized that dose to medium is the long‐term goal of treatment planning solutions and most treatment planning dose calculation engines now provide something as close as possible to this.

The Versa HD accelerator head is modeled in this work, as this is the most widely used accelerator at this center, but the model is sufficiently general to be applied to other accelerators. Schach von Wittenau et al.^7^, ^8^ show good agreement between a computational phase space and a full Monte Carlo simulation of the beam for 600C and 2100C linear accelerators (Varian). The work of Fix et al.^14^, ^15^, ^16^ is centered on Varian Clinac accelerators, and also shows good agreement between simple source models and full Monte Carlo simulation. Meanwhile, Nwankwo et al.^13^ model the Synergy accelerator (Elekta), which is similar to that used in the present study. Aboulbanine et al.^17^ compare the phase space produced by a virtual source model with the standard phase space data provided by the International Atomic Energy Authority (IAEA),^39^ with moderately good agreement for the 6 and 10 MV beams of a Precise accelerator (Elekta) and for the 6 MV beam of a Truebeam accelerator (Varian). They also demonstrate good agreement between dose calculations resulting from the virtual source model and from the standard phase space, when using GEANT4 as the Monte Carlo engine. Their work^17^ is for rectangular fields, and a subsequent report^18^ describes the incorporation of a multileaf collimator into the virtual source model.

Compared to these studies, the method in the current paper has the advantage of being related to a clinically commissioned commercial treatment planning system. As the parameters in the phase space model relate closely to those in the treatment planning system, it is possible to generate a phase space model for application to clinical treatment plans with maximum efficiency. Some manual adjustment of the beam parameters is still necessary, but the required changes are intuitive and can be manually applied. Generally, the other studies in the literature^7^, ^8^, ^13^, ^14^, ^15^, ^16^ described above, compare a source model with a full phase space, thus achieving closer agreement than when comparing a source model with another dose calculation algorithm, as in the present study.

The work described is expected to form the basis of a dose reconstruction method for application to real‐time adaptive radiotherapy. The fine phase space and dose grid are chosen in this study for optimal accuracy, and give rise to a computation that is too slow to be used in real time. However, with careful adjustment of these parameters, real time calculations may be possible. For example, it may be useful to reduce the resolution of the phase space grid and to use a slightly coarser dose grid. Reducing the number of particle histories while increasing the final filtering is another area of practical interest. For example, Bai et al.^40^ use a machine learning technique to de‐noise a Monte Carlo dose distribution generated using very few particle histories.

A number of authors describe the use of a graphics processing unit (GPU) to increase the parallelism of the computation.^41^ This approach is pursued by Jia et al., who describe the implementation of the DPM code on GPU, with one to two orders of magnitude speedup compared to a single‐thread implementation.^42^, ^43^ Townson et al.^44^ describe simplified phase‐space models for this implementation, so as to avoid the time overhead associated with reading a large phase‐space file. GPU implementations of the GEANT4 and PENELOPE codes are also described in the literature,^45^, ^46^ as well as new ground‐up codes specifically intended for GPU.^47^ One of the difficulties of implementing Monte Carlo calculation on GPU is that the progress of the calculations on different units can diverge with time due to differences in calculation efficiency. Rejection sampling contributes significantly to this effect, and Liang et al. therefore replace rejection sampling with inverse transform sampling.^48^

In the meantime, multi‐core CPU architectures have progressed, so that calculation of 100 threads on CPUs is possible, giving a computation speed which may be competitive with GPU implementations. Whichever method is chosen for computation, there is scope to improve the calculation speed by several orders of magnitude, opening up the possibility of real‐time calculation. Such real‐time application is an interesting and potentially valuable aspect to the use of Monte Carlo simulation in radiotherapy.

## 5 CONCLUSIONS

A simple dual‐source accelerator head model can be used successfully to construct a phase space for application to fast Monte Carlo dose calculation. The parameters in this study are derived from the clinical RayStation beam model used for convolution dose calculation at this center, with minimal adjustments required. When the phase space is applied to the DPM Monte Carlo dose calculation code, good agreement with dose calculated by the convolution algorithm in RayStation is obtained. There is therefore scope for application of the phase space model to Monte Carlo calculation in a real‐time adaptive context.

## CONFLICT OF INTEREST

The authors declare no conflict of interest.

## AUTHOR CONTRIBUTIONS

James L Bedford: study design; collection of data. Rahul Nilawar: software design. Simeon Nill: conception of study. Uwe Oelfke: conception of study. All authors additionally critically revised the manuscript and approved the final version, and agree to be accountable for the accuracy of the work.
